# Infections and risk factors for infection-related mortality after pediatric allogeneic hematopoietic stem cell transplantation in Mexico: A single center retrospective study

**DOI:** 10.1371/journal.pone.0284628

**Published:** 2023-09-29

**Authors:** Elva Jiménez-Hernández, Juan Carlos Núñez-Enriquez, José Arellano-Galindo, María de los Angeles Del Campo-Martínez, Perla Verónica Reynoso-Arenas, Alfonso Reyes-López, Alejandra Viridiana Delgado-Gaytan, María Del Socorro Méndez-Tovar, Teresa Marín-Palomares, María Teresa Dueñas-Gonzalez, Antonio Ortíz-Fernández, Inés Montero-Ponce, Laura Eugenia Espinosa-Hernández, Nora Nancy Núñez-Villegas, Ruy Pérez-Casillas, Berenice Sánchez-Jara, Angel García-Soto, Annecy Nelly Herver-Olivares, Ethel Zulie Jaimes-Reyes, Hector Manuel Tiznado-García, Octavio Martínez-Villegas, Betzayda Valdez-Garibay, Paloma Del Rocío Loza-Santiaguillo, Xochiketzalli García-Jiménez, Guadalupe Ortíz-Torres, Gabriela Jazmin Fernández-Castillo, Dulce María Aguilar-Olivares, Luis Alejandro Díaz-Padilla, Mario Alberto Noya-Rodríguez, Mariana García-Jiménez, Juan Manuel Mejía-Aranguré

**Affiliations:** 1 Servicio de Hematología Pediátrica y Unidad de Trasplante de Médula Osea, Unidad Médica de Alta Especialidad (UMAE), Centro Médico Nacional (CMN)” La Raza”, Instituto Mexicano del Seguro Social (IMSS), Mexico City, Mexico; 2 Laboratorio de Genómica del Cáncer, Instituto Nacional de Medicina Genómica, Mexico City, Mexico; 3 Unidad de Investigación Médica en Epidemiología Clínica, UMAE, Hospital de Pediatría, CMN “Siglo XXI”, IMSS, Mexico City, Mexico; 4 Laboratorio de Virología Clínica y Experimental, Unidad de Investigación Unidad de Investigación en Enfermedades Infecciosas, Hospital Infantil de México “Federico Gómez” Ciudad de México, México, Mexico; 5 Servicio de Hematología Pediátrica, UMAE, CMN “La Raza” del IMSS, Mexico City, Mexico; 6 Centro de Estudios Económicos y Sociales en Salud, Hospital Infantil de México Federico Gómez, de la Secretaría de Salud, México City, Mexico; 7 Laboratorio Clínico, Sección de Microbiología, UMAE, CMN “La Raza” del IMSS, Mexico City, Mexico; 8 Centro Estatal de Cancerología, “Dr. Miguel Dorantes-Mesa”, Secretaría de Salud, Xalapa Veracruz, México; 9 Hospital Pediátrico Coyoacán, Secretaría de Salud Gobierno de la Ciudad de México, Mexico City, México; 10 Facultad de Estudios Superiores Iztacala, Universidad Nacional Autónoma de México, Mexico City, Mexico; 11 Facultad de Medicina, Universidad Nacional Autónoma de México (UNAM), Mexico City, Mexico; Shiraz University of Medical Sciences, ISLAMIC REPUBLIC OF IRAN

## Abstract

**Objective:**

To identify the type of infections and risk factors for infection-related mortality (IRM) after allogeneic hematopoietic stem cell transplantation (HSCT).

**Methods:**

Retrospective cohort study of patients <16 years of age treated in 2010–2019 was conducted. Unadjusted hazard ratios (HR) and adjusted hazard ratios (aHR) with 95% confidence intervals (95% CIs) were estimated using Cox regression. Cumulative incidence was calculated.

**Results:**

Data for 99 pediatric patients were analyzed. The myeloablative conditioning was the most used regimen (78.8%) and the hematopoietic stem cell source was predominantly peripheral blood (80.8%). Primary graft failure occurred in 19.2% of patients. Frequency of acute graft-versus-host disease was 46.5%. Total of 136 infectious events was recorded, the most common of which were bacterial (76.4%) followed by viral infection (15.5%) and then fungal infection (8.1%). The best predictors for infection subtypes where the following: a) for bacterial infection (the age groups of 10.1–15 years: aHR = 3.33; 95% CI: 1.62–6.85 and. >15 years: aHR = 3.34; 95% CI: 1.18–9.45); b) for viral infection (graft versus host disease: aHR = 5.36; 95% CI: 1.62–17.68), however, for fungal infection statistically significant predictors were not identified. Related mortality was 30% (n = 12). Increased risk for infection-related mortality was observed in patients with unrelated donor and umbilical cord stem cells recipients (HR = 3.12; 95% CI: 1.00–9.85).

**Conclusions:**

Frequencies of infections and infection-related mortality appear to be similar to those reported. Unrelated donors and stem cells from umbilical cord recipients were associated with a high risk of mortality.

## Introduction

Hematopoietic stem cell transplantation (HSCT) is a well-established treatment modality intended to cure and/or improve a variety of hematological, oncological, hereditary, and immunological diseases [1, 2]. Its efficacy has improved significantly over the past 20 years, but infections after transplantation still contribute to high rates of complications and deaths [2, 3]. The frequency and type of infections after HSCT in the pediatric population depend on several factors such as the graft phase, type of microorganism, hospital center, age at the time of transplantation, and restitution of the immune system functionality [3–10]. For instance, infections by Gram-negative bacteria, Gram-positive bacteria, and fungi such as *Candida albicans* are more common during the early or pregraft phase as a consequence of the development of severe neutropenia, the breakdown of natural barriers and the placement of central venous catheters [11, 12]. Moreover, invasive Aspergillosis, the most common fungal infection during the immediate and late postgraft periods ais related to the occurrence of graft-versus-host disease (GVHD) and the prolonged use of steroids [13–15]. *Pneumocystis jirovecii* is another fungus that appears in the later stages of transplantation [16]. On the other hand, the herpes viruses and cytomegalovirus (CMV) are the most common viral infections occurring during the postgraft phase [17], particularly, in patients who were positive before transplantation [18].

The contributing factors that have been described for the development of infections after HSCT include recipient demographics, neutropenia, transplantation modality, donor type, human leukocyte antigen (HLA) compatibility, conditioning regimen, the origin of hematopoietic cells, and donor/recipient (D/R) CMV serologic status [19, 20]. Then, the complex interaction among these elements makes it difficult to identify the specific risk factors for the development of bacterial, fungal, and viral infections and infection-related mortality (IRM) after HSCT.

In developed countries, the control of infectious complications after HSCT has improved progressively through the development of molecular methods for the detection of viral and fungal infections, the use of preemptive treatment, the introduction of new antifungal agents, and the implementation of strategies for the prevention of nosocomial infections, all of which have reduced mortality over time [21–23]. In Mexico, there are limited data on the frequency and type of infections and infection-related mortality after allogeneic HSCT in pediatric patients. Collecting such data may help to improve supportive care and reduce mortality in this population. The present study aimed to identify the types of infections and risk factors for IRM after pediatric allogeneic HSCT.

## Patients and methods

A retrospective cohort study was conducted in the Bone Marrow Transplant Unit of the National Medical Center “La Raza,” Mexican Institute of Social Security, located in Mexico City. All patients <16 years old who underwent their first allogeneic HSCT during the study period (January 2010 to December 2019) were included in the current investigation. Patients were hospitalized in a single closed unit room with filtered air and conditions as previously described [24].

To ensure the confidentiality of the participants a consecutive identification number was used in the database instead of the name or other personally identifiable information. All the procedures of the bone marrow transplantation program described here were approved by the Ethics, Biosafety, Mortality, and Transplantation Institutional Committees. Before the procedure, written consent inform was signed by the parents of each child. The present research followed the Equator STROBE (Strengthening the Reporting of Observational studies in Epidemiology) guidelines recommended for reporting observational studies [25].

Clinical information was obtained through the review of clinical records. To validate the information, the prospectively updated records in the transplant unit were reviewed along with the database of cultures for bacteria, viruses, and fungi in the Clinical Microbiology Laboratory.

### Definitions

Febrile neutropenia: The increase in body temperature to >38.5°C once or 38–38.5°C twice in a 4-hour period; associated with severe neutropenia (<500/μL); no microorganism growth in cultures was observed; and empirical treatment was implemented [19].

Bacterial infection: Invasion and multiplication by pathogenic bacteria visualized in blood cultures after HSTC. Colonies had grown and were identified as Gram-positive or -negative bacteria; the genus and species, as well as antibiotic-sensitivity profile, were established using standardized methods [11].

Fungal infection: Invasion and multiplication by pathogenic fungi visualized in cultures after HSCT. Yeasts or hyphae were identified according to their morphological characteristics. Biochemical tests were used to identify the genus and species and to determine the sensitivity or resistance to antifungal agents. The galactomannan test was used to detect invasive aspergillosis. The definition of invasive fungal infection followed the consensus of the EORTC/MSG (European Organization for Research and Treatment of Cancer/ Mycoses Study Group) [13].

Active viral infection: Detected by real-time polymerase chain reaction (RT-PCR) of viral Desoxyribonucleic Acid (DNA) in fluids (blood, saliva, bronchoalveolar lavage, urine) performed weekly after HSCT for CMV, Epstein–Barr virus (EBV), BK (Polyomavirus), and herpes simplex virus (HSV) types I and II. A positive test was interpreted to indicate invasion and multiplication by pathogenic viruses for monitoring treatment effectiveness and was the basis for the decision to start preemptive therapy [21].

Episode of infection. It was defined as the entrance, develop and multiplication of an infectious agent (bacterial, virus or fungus) in the post-transplantation phase, which was resolved in a period less than 14 days with the habitual treatment.

### Anti-infective prophylaxis

During the study period, two types of anti-infective prophylaxis schemes were used based on the availability of new antimicrobial agents in the Institution. The first scheme that was used consisted in the administration of ciprofloxacin, fluconazole, and acyclovir. The second and currently used scheme consists in cefepime, voriconazole and acyclovir. The doses, intervals, and duration are following described:

Ciprofloxacin was administered at 10 mg/kg IV or per oral (PO) every 12 hours from the start of conditioning until engraftment. It was substituted at the emergence of fever and/or at the moment that a microorganism non-sensitive to this drug was identified. Additionally, it was used during the post-graft phase until completion of the first 100 days and it was continued in case of GVHD.

Cefepime was given at doses of 50 mg/kg intravenous (IV) every 12 hours in children under 40kg. In patients weighting >40kg it was administered at 2gr IV every 12 hours from the start of conditioning until engraftment. It was substituted at the emergence of fever and/or at the moment that a microorganism non-sensitive to cefepime was identified. the time of patient discharge it was substituted by ciprofloxacin 10 mg/kg PO every 12 hours.

Fluconazole was administered at a dose of 6–12 mg/kg/day (maximum 400 mg/day) IV or PO from the start of conditioning until engraftment. It was continued in case of GVHD until the immune reconstitution.

Voriconazole was given as an impregnation dose of 6mg/kg IV every 12 hours for 2 doses and the maintenance dose consisted in 4mg/kg every 12 hours from the start of conditioning until engraftment. At hospital discharge, it was continued at doses of 100mg PO every 12 hours in patients between 2 and 11 years of age (< 40kg) and 200 mg PO every 12 hours in patients older than 11 years (>40 kg). Furthermore, it was used during the post-graft phase until completion of the first 100 days and it was continued in case of GVHD until the immune reconstitution and/or in case of a galactomannan test resulted positive.

Prophylaxis for *Pneumocystis jirovecii* was done with trimethoprim /sulfamethoxazole at a dose 5mg/20mg/kg/day PO during 2 consecutive days twice per week during 6 months. It was continued only in patients with GVHD until the immune reconstitution, this was started once the engraftment was successful whereas

Antiviral prophylaxis was done with acyclovir 20 mg/kg/day three times a day (every 8 hours) from the first pre-engraftment stage to 365 d post-transplantation and ones the immune reconstitution was reached as well as the immunosuppression was suspended. In cases with suspicion of resistance to acyclovir, this was substituted for valacyclovir 15–30 mg/kg/day three times a day (every 8 hours).

Pre-emptive treatment was started taking into consideration a CMV viral load >1000 copies/mL in whole blood on two measurements. It consisted of ganciclovir 5 mg/kg IV every 12 hours. The treatment was continued for 2 weeks or until CMV viral load was <1000 copies/mL.

### Laboratory procedures

#### Hemocultures

Hemocultures were done by the use of Ped BacT method. Briefly: Four mL were collected from each patient by venopunction with strict asepsis and immediately inoculated in a Pedi BactT bottle, each bottle was incubated in the System and monitored automatically every 10 minutes, a positive test was considered when the production of CO2 was detected in the culture medium, this was done automatically when a change of color from blue to green and finally yellow in the bottom of the tube is detected by the system. A negative test was considered when the culture reached 5 days of incubation without detection of color changes by the system.

#### Galactomannan test

This was performed in serum from the patient by the use of the kit Platelia *Aspergillus* EIA Cat. 72696 Bio Rad® (Marnes-la-Coquette France) following the instructions of the manufacturer. Briefly: serum was heated to 120°C/ 6 min in a dried block, and then processed in an immunoenzymatic sandwich system, the serum sample is added to wells coated with a Mab anti galactomannan and then a second conjugate antibody-peroxidase was added and revealed with the respective substrate. A blue color was developed when galactomannan was detected in the serum sample, the index was calculated and the cut-off was established according to the manufacturer as follows: a sample serum with an index ≥0.5 was considered positive whereas a sample serum with an index <0.5 was considered negatives. In those cases were the first test was positive, the patient was monitored each week and the result was correlated with the clinical response until the galactomannan test was negative.

#### Viral load

Viral load for CMV was done weekly by the use of the kits Abbot Real time for CMV assay (ref 09N21-090, Des Plaines, Illinois, USA) which have a sensitivity of 20 copies/ml in plasma sample.

### Ethical considerations

The research protocol was approved by the Scientific and Ethics Committee of the National Medical Center “La Raza,” (registration number 2020-3502-029). All the applicable regulatory requirements and ethical principles were fulfilled.

### Statistical analysis

Data were analyzed using Stata program version 16 (StataCorp 4905 Lakeway Drive College Station, Texas 77845 USA). Initially, a descriptive analysis was performed to present the frequency distribution of the categorical variables. The results are expressed in absolute numbers and percentages. The median and range were calculated for continuous variables. Cox (Proportional Hazards) Regression analyses were used for calculating unadjusted and adjusted hazard ratios (HR and aHR, respectively). 95% confidence intervals were used to report the effect size precision estimations. Additionally, a competing risk analysis was conducted considering the IRM as the event of interest and the relapse or death (different from IRM) as the competing events, based on the method of Fine and Gray (1999) [26], which provides us with a useful alternative to Cox regression for survival data in the presence of competing risks.

The study variables were gender, age (in years), age group (0–5, 5.1–10, 10.1–15, and >15 years), donor type (related/unrelated), the origin of hematopoietic stem cells (HSCs) (umbilical cord/peripheral blood), conditioning regimen (myeloablative [MA]/non-MA), graft failure (yes/no), CMV serological status of the recipient and donor (negative/positive), type of anti-infective prophylaxis, and GVHD (yes/no). The outcomes were bacterial infection, fungal infection, viral infection, and infection-related mortality (IRM). The database used for the present analysis is attached as S1 Data.

## Results

### Patient demographic data

During the study period a total of 99 pediatric patients underwent allogeneic HSCT and were included in the present research. The population included a higher frequency of boys (62.6%). The median age at the time of transplantation was 8 years (range: 1–16 years). The most frequent age range was 5.1–10 years (36.4%) followed by 10.1–15 years (30.3%). The most common diagnosis that led to allogeneic HSCT was acute lymphoblastic leukemia (57.6%), followed by bone marrow failure (17.2%) and some other less common diagnoses (6.1%) (Table 1).

**Table 1:** General characteristics of pediatric patients who underwent HSCT during the study period.

Variables	Patients (n = 99)	
	n (%)	Median (min-max)
**Gender**		
Male	62 (62.6)	
Female	37 (37.4)	
**Age (years)**		8 (1–16)
**Age groups (years)**		
0–5	26 (26.3)	
5.1–10	36 (36.4)	
10.1–15	30 (30.3)	
>15	7 (7.1)	
**Diagnoses**		
Acute lymphoblastic leukemia (ALL)	57 (57.6)	
Acute myeloid leukemia (AML)	5 (5.1)	
Bone marrow failure	17 (17.2)	
Lymphoma	5 (5.1)	
Myelodysplastic syndromes	5 (5.1)	
Immunodeficiencies	4 (4.0)	
Other	6 (6.1)	
**Follow-up time from HSCT (days)**	** **	585 (15–3757)

Abbreviations: n, number of patients; min, minimum; max, maximum

S1 Table displays the characteristics of the transplant procedure. The most frequent conditioning regimen was MA (78.8%), which was based on the combination of busulfan and cyclophosphamide, and the non-MA regimen comprised cyclophosphamide with anti-thymocyte globulin. The most frequent donor types were the related type in 80.8% (n = 80) and the identical type according to HLA compatibility (10/10). Hematopoietic stem cells (HSCs) were obtained mainly from peripheral blood (80.8%; n = 80).

The predominant prophylactic scheme to prevent GVHD comprised cyclosporine, mycophenolate mofetil, and cyclophosphamide (33.3%), followed by cyclosporine and mycophenolate mofetil (22.2%). Anti-infective prophylaxis was based mainly on cefepime, voriconazole, and acyclovir (80.8%). The CMV serology status was similar between recipients and donors (84.8 and 85.9% positive, respectively). Information about the outcomes of the transplant procedure is shown in the S2 Table.

Primary graft failure was found in 19.2% (n = 19) of patients, 12 of whom (70.6%) had been transplanted with umbilical cord blood and the other five (29.4%) were haploidentical patients transplanted with peripheral blood. Secondary graft failure was mainly a consequence of disease relapse (52.5%). Acute GVHD occurred in 46.5% (n = 46) of patients and was mainly grade 1 (58.7%, n = 27) with the skin as the most affected site (50%, n = 23). Chronic GVHD occurred in 12.8% of patients (n = 12).

### Frequency of infections

Febrile neutropenia occurred in 41.4% (n = 41) of patients. The median time when this event was registered after HSCT was 6 days (IQR: 1–17 days). The number of infection episodes by the type of microorganism, time of occurrence, and isolation sites are shown in Table 2. Of the 99 transplanted patients, a total of 136 episodes of infection were recorded: 104 (76.4%) events were caused by bacterial infections, 11 (8.1%) due to fungal infections, and 21(15.5%) were related to viral infections.

**Table 2:** Number of episodes of infection by type of microorganism, time of occurrence, and isolation site (total episodes = 136).

	Microorganism and site	Number of episodes (%)	Median (min-max)
**Bacterial infections**	**104**	**(76.4)**	
Gram-positive	**52**	(38.2)	
*Staphylococcus epidermidis*	35	(25.7)	
*Enterococcus faecalis*	4	(2.9)	
*Enterococcus faecium*	6	(4.41)	
*Staphylococcus aureus*	4	(2.9)	
*Staphylococcus hominis*	3	(2.2)	
Gram-negative	**38**	**(36.5)**	
*Escherichia coli*	27	(19.8)	
*Pseudomonas aeruginosa*	4	(2.9)	
*Enterobacter cloacae*	3	(2.2)	
*Klebsiella pneumonia*	2	(1.4)	
*Stenotrophomonas maltophilia*	2	(1.4)	
Other	14	(13.5)	
Time of bacterial infection occurrence after HSCT (days)			**11.5 (1–57)**
Isolation site			
Bloodstream	36	(34.6)	
Urine	27	(25.9)	
Peripheral/Central Venous Catheters	26	(25.0)	
Skin (abscess, cellulitis)	5	(4.8)	
Other	10	(9.6)	
**Fungal infections**	**11**	**(8.1)**	
*Candida albicans*	4	(2.9)	
*Candida guilliermondii*	2	(1.4)	
*Candida tropicalis*	2	(1.4)	
*Aspergillus fumigatus*	2	(1.4)	
*Aspergillus brasiliensis*	1	(0.7)	
Time of fungal infection occurrence after HSCT (days)			**43 (18–218)**
Bloodstream	4	(2.9)	
IS*			
Lung	4	(2.9)	
Other	3	(2.1)	
**Viral infections**	**21**	**(15.5)**	
Cytomegalovirus	11	(7.9)	
BK virus	3	(2.2)	
Herpes zoster	3	(2.2)	
Epstein–Barr	2	(1.4)	
Other	2	(1.4)	
Time of viral infection occurrence after HSCT (days)			**70 (25–365)**
Blood			
IS*			
Urine			
Bronchoalveolar lavage fluid	3	(2.2)	
Skin	4	(2.9)	
Other	2	(1.4)	

Abbreviations: min, minimum; max, maximum; HSCT, hematopoietic stem cell transplantation

IS*, isolation site.

The main sites from which microorganisms causing bacterial infections were isolated were the bloodstream (34.6%), urine (25.9%), and peripheral/central venous catheters (25%). Gram-positive bacteria represented 38.2% of all bacterial infections. The most frequently identified bacteria were the Staphylococcus epidermidis (25.7%) and Escherichia coli (19.8%). The median time when these infections occurred was 11.5 days (IQR: 1–57 days). The median time after transplant when fungal infections occurred was 43 days (IQR: 18–218 days). *Candida albicans* was the most frequently identified fungus. The median time after transplant when viral infection occurred was 70 days (IQR: 25–365 days). Among the most frequently detected viruses were CMV (7.9%), BK virus (2.2%), and Herpes zoster (2.2%).

### Factors associated with a bacterial infection

Table 3 contains the results of univariate and multivariate analysis with Cox regression for the identification of factors associated with the development of bacterial infections after allogeneic HSCT.

**Table 3:** Cox regression analyses for identifying the factors associated with bacterial infections in pediatric patients after allogeneic HSCT (n = 99).

Study variable	Bacterial infection					
	No	Yes		HR (95% CI)	aHR (95% CI)	p-value
	n = 33	n = 66				
	n (%)	n (%)				
**Gender**						
Female	12 (36.4)	25 (37.9)		—	—	
Male	21 (63.6)	41 (62.1)		0.92 (0.56–1.52)	0.77 (0.45–1.31)	0.35
**Age groups (years)**						
0–5	11(33.3)	15 (22.7)		—	—	
5.1–10	15 (45.5)	21 (31.8)		1.28 (0.66–2.49)	1.58 (0.76–3.28)	0.22
10.1–15	6 (18.2)	24 (36.4)		2.60 (1.35–4.99)	3.33 (1.62–6.85)	<0.01
>15	1 (3)	6 (9.1)		2.38 (0.92–6.14)	3.34 (1.18–9.45)	0.02
**Donor type**						
Related	29 (87.9)	51 (77.3)		—	—	
Unrelated	4 (12.1)	15 (22.7)		1.21 (0.68–2.15)	1.45 (0.74–2.84)	0.27
**Origin of hematopoietic stem cells**						
Peripheral blood	29 (87.9)	51 (77.3)		—	—	
Umbilical cord	4 (12.1)	15 (78.9)		1.21 (0.68–2.15)	NC	NC
**Conditioning regimen**						
Nonmyeloablative	4 (12.1)	17 (25.7)		—	—	
Myeloablative	29 (87.9)	49 (74.3)		0.65 (0.37–1.14)	0.68 (0.37–1.26)	0.23
**Graft failure**						
No	25 (75.8)	53 (80.3)		—	—	
Yes	8 (24.2)	13 (18.2)		0.77 (0.42–1.41)	0.75 (0.38–1.51)	0.43
**CMV serological status of the recipient (pretransplant)**						
Negative	7 (21.2)	8 (12.1)		—	—	
Positive	26 (78.8)	58 (87.9)		1.65 (0.78–3.47)	1.72 (0.78–3.78)	0.18
**Anti-infective prophylaxis**						
Cefepime, voriconazole, acyclovir	30 (90.9)	51(77.3)		—	—	
Ciprofloxacin, fluconazole, acyclovir	3 (9.1)	15 (22.7)		1.45 (0.81–2.59)	1.77 (0.89–3.53)	0.10
**Graft-versus-host disease**						
No	17 (51.5)	36 (54.5)		—	—	
Yes	16 (48.5)	30 (45.5)		0.93 (0.57–1.52)	0.81 (0.47–1.42)	0.47

Note: Cox (Proportional Hazards) Regression analyses were used for calculating HR and aHR.

Abbreviations: HR, unadjusted hazard ratio; aHR, adjusted hazard ratio; 95% CI, 95% confidence interval; NC, not calculated because of insufficient sample size;; *p-value for adjusted HRs.

The MA conditioning regimen was associated with a low risk of bacterial infections; however, the result was not statistically significant. Remarkably, at greater the age at transplantation was, the greater the risk of bacterial infection. Particularly, in the age groups of 10.1–15 years (aHR = 3.33; 95% CI: 1.62–6.85) and. >15 years (aHR = 3.34; 95% CI: 1.18–9.45). The presence of GVHD did not influence the risk of bacterial infection (aHR = 0.81; 95% CI: 0.47–1.42).

### Factors associated with fungal infection

The age and gender were similar between patients with and without fungal infections (Table 4). Regarding the donor type, an increased risk for fungal infections was noted in the unrelated group, however, given the limited sample size, no statistically significant association was observed. The anti-infective prophylaxis which included fluconazole as the anti-fungal medication showed a higher risk for developing fungal infections compared with the prophylaxis scheme that included voriconazole, nonetheless, the association was not statistically significant (aHR = 1.88; 95% CI: 0.43–8.10). A similar trend to increase in the risk for fungal infections was observed in patients who presented GVHD but no statistically significant results were obtained.

**Table 4:** Cox regression analyses for identifying the factors associated with fungal infections in pediatric patients after allogeneic HSCT (n = 99).

Study variables	Fungal infections				
	No	Yes	HR (95% CI)	aHR (95% CI)	p-value*
	n = 88	n = 11			
	n (%)	n (%)			
**Gender**					
Female	33 (37.5)	4 (36.4)	—	—	
Male	55 (62.5)	7 (63.6)	1.00 (0.29–3.41)	1.07 (0.27–4.16)	0.91
**Age groups (years)**					
0–5	22 (25)	4 (36.4)	—	—	
5.1–10	33 (37.5)	3 (27.3)	0.49 (0.11–2.19)	0.54 (0.10–2.76)	0.46
10.1–15	27 (30.7)	3 (27.3)	0.66 (0.14–2.95)	0.76 (0.15–3.81)	0.74
>15	6 (6.8)	1 (9)	0.96 (0.10–8.67)	1.63 (0.14–18.56)	0.69
**Donor type**					
Related	73 (82.9)	7 (63.6)	—	—	
Unrelated	15 (17.1)	4 (36.4)	2.51 (0.73–8.62)	3.52 (0.74–16.75)	0.11
**Origin of hematopoietic stem cells**					
Peripheral blood	73 (82.9)	7 (63.6)	—	—	
Umbilical cord	15 (17.1)	4 (36.4)	2.51 (0.73–8.62)	NC	NC
**Conditioning regimen**					
Non-myeloablative	19 (21.6)	2 (18.2)	—	—	
Myeloablative	69 (78.4)	9 (81.8)	1.15 (0.25–5.35)	1.58 (0.24–10.34)	0.63
**Graft failure**					
No	70 (79.5)	8 (72.7)	—	—	
Yes	18 (20.5)	3 (27.3)	1.36 (0.36–5.16)	1.46 (0.29–7.26)	0.64
**CMV serological status of the recipient (pretransplant)**					
Negative	14 (15.9)	1 (9.1)	—	—	
Positive	74 (84.1)	10 (90.9)	1.93 (0.24–15.14)	2.09 (0.22–19.66)	0.52
**Anti-infective prophylaxis**					
Cefepime, voriconazole, acyclovir	14 (15.9)	4 (36.4)	—	—	
Ciprofloxacin, fluconazole, acyclovir	74 (84.1)	7 (63.6)	2.86 (0.83–9.83)	1.88 (0.43–8.10)	0.40
**Graft-versus-host disease**					
No	50 (56.8)	3 (27.3)	—	—	
Yes	38 (43.2)	8 (72.7)	3.16 (0.84–11.93)	3.36 (0.78–14.40)	0.10

Note: Cox (Proportional Hazards) Regression analyses were used for calculating HR and aHR.

Abbreviations: HR, unadjusted hazard ratio; aHR, adjusted hazard ratio; 95% CI, 95% confidence interval; NC, not calculated because of insufficient sample size

*p-value for adjusted HRs.

### Factors associated with a viral infection

The only statistically significant risk factor for viral infection was the development of GVHD (aHR = 5.36; 95% CI: 1.62–17.68; p<0.01) (Table 5). Notwithstanding, the frequency of viral infection was higher in boys (68.4%; n = 13) and the 5.1–10-year age group (26.3%) as well as in the 10.1–15 years age group, however, these associations were not statistically significant. In addition, the frequency of viral infection was much higher in patients who were CMV+ before the transplant (94.7%, n = 18) than in those who were CMV–(5.3%, n = 1), but this difference was not significant presumably because of the limited sample size (aHR = 4.66; 95% CI: 0.54–39.89; p = 0.16.

**Table 5:** Cox regression analyses for identifying the factors associated with viral infections in pediatric patients after allogeneic HSCT (n = 99).

Study variables	Viral infections				
	No	Yes	HR (95% CI)	aHR (95% CI)	p-value
	n = 80	n = 19			
	n (%)	n (%)			
**Gender**					
Female	31 (38.8)	6 (31.6)	—	—	
Male	49 (61.2)	13 (68.4)	1.23 (0.46–3.23)	1.19 (0.40–3.55)	0.75
**Age groups (years)**					
0–5	19 (23.8)	7 (36.9)	—	—	
5.1–10	31 (38.8)	5 (26.3)	0.46 (0.14–1.45)	0.31 (0.09–1.08)	0.06
10.1–15	25 (31.2)	5 (26.3)	0.65 (0.20–2.07)	0.54 (0.16–1.86)	0.34
>15	5 (6.2)	2 (10.5)	1.14 (0.23–5.49)	1.79 (0.31–10.25)	0.51
**Donor type**					
Related	66 (82.5)	14 (73.7)	—	—	
Unrelated	14 (17.5)	5 (26.3)	1.52 (0.54–4.23)	1.14 (0.33–3.94)	0.83
**Origin of hematopoietic stem cells**					
Peripheral blood	66 (82.5)	14 (73.7)	—	—	
Umbilical cord	14 (17.5)	5 (26.3)	1.52 (0.54–4.23)	NC	NC
**Conditioning regimen**					
Non-myeloablative	15 (18.8)	6 (31.6)	—	—	
Myeloablative	65 (81.2)	13 (68.4)	0.52 (0.19–1.37)	0.42 (0.13–1.40)	0.16
**Graft failure**					
No	61 (76.2)	17 (89.5)	—	—	
Yes	19 (23.8)	2 (10.5)	0.42 (0.09–1.84)	0.49 (0.09–2.45)	0.39
**CMV serological status of the recipient (pretransplant)**					
Negative	14 (17.5)	1 (5.3)	—	—	
Positive	66 (82.5)	18 (94.7)	3.75 (0.50–28.11)	4.66 (0.54–39.89)	0.16
**Anti-infective prophylaxis**					
Cefepime, voriconazole, acyclovir	13 (16.3)	5 (26.3)	—	—	
Ciprofloxacin, fluconazole, acyclovir	67 (83.7)	14 (73.7)	1.70 (0.61–4.76)	1.14 (0.32–4.08)	0.83
**Graft-versus-host disease**					
No	48 (60)	5 (26.3)	—	—	
Yes	32 (40)	14 (73.7)	3.63 (1.30–10.10)	5.36 (1.62–17.68)	<0.01

Note: Cox (Proportional Hazards) Regression analyses were used for calculating HR and aHR.

Abbreviations: HR, unadjusted hazard ratio; aHR, adjusted hazard ratio; 95% CI, 95% confidence interval; NC, not calculated because of insufficient sample size;; *p-value for adjusted HRs.

### Causes of death and infection-related mortality after HSCT

A total of 40 patients died after HSCT, giving an overall survival rate of 59.6%. The median follow-up time was 250 days (IQR: 81–2,045 days). The main cause of death was relapse/progression of the underlying disease (52.5%, n = 21).

The second cause was infection-related mortality (IRM) (30%; n = 12). The remaining causes of mortality (17.5%) (not related to infections) were a consequence of the following: graft failure, GVHD, hemorrhage, thrombosis, and brain death, either alone or combined with infections. The specific type of infections, complications and outcomes after the development of infections are shown in S3 Table.

Noteworthy, those patients who received a MA conditioning regimen had a lower risk for IRM in comparison to children where a non-MA regimen was used, however, this result was non-statistically significant (aHR = 0.34; 95% CI: 0.10–1.19). By contrast, in patients who had CMV-positive results pretransplant, a trend for an increased risk of infection-related mortality was noted (HR = 2.12; 95% CI: 0.27–16.44) (Table 6).

**Table 6:** Association between study variables and infection-related mortality in the population (n = 99).

Study variables	HR (95% CI)	aHR (95% CI)	p-value*
**Gender**			
Female	—	—	
Male	1.13 (0.34–3.78)	0.74 (0.17–3.08)	0.68
**Age group (years)**			
0–5	—	—	
5.1–10	0.35(0.65–1.94)	0.47 (0.07–2.98)	0.42
10.1–15	1.22 (0.32–4.55)	2.06 (0.43–9.85)	0.37
>15	1.11 (0.12–9.97)	2.87 (0.24–34.30)	0.4
**Donor type**			
Related	—	—	
Unrelated	3.12 (1.00–9.85)	2.72 (0.60–12.35)	0.19
**Origin of hematopoietic stem cells**			
Peripheral blood	—	—	
Umbilical cord	3.12 (1.00–9.85)	NC	NC
**Conditioning regimen**			
Non-myeloablative	—	—	
Myeloablative	0.25 (0.08–0.80)	0.34 (0.10–1.19)	0.09
**Graft failure**			
No	—	—	
Yes	0.035 (0.00–15.2)	NC	NC
**CMV serological status of the recipient (pretransplant)**			
Negative	—	—	
Positive	2.12 (0.27–16.44)	2.43 (0.27–21.44)	0.42
**Anti-infective prophylaxis**			
Cefepime, voriconazole, acyclovir	—	—	
Ciprofloxacin, fluconazole, acyclovir	1.64 (0.44–6.08)	1.03 (0.19–5.62)	0.97
**Graft-versus-host disease**			
No	—	—	
Yes	1.62 (0.51–5.12)	1.94 (0.50–7.40)	0.33

Note: Cox (Proportional Hazards) Regression analyses were used for calculating HR and aHR.

Abbreviations: HR, unadjusted hazard ratio; aHR, adjusted hazard ratio; 95% CI, 95% confidence interval; NC, not calculated because of insufficient sample size

*p-value for adjusted HRs.

### Competing-risk analysis results

A total of 93 subjects with complete data entered the model, of which 11 experienced IRM, 29 experienced relapse or death (different from IRM), and the remaining 53 were event-free cases. The results of the competing-risks model are displayed in Table 7.

**Table 7:** Results of the competing-risks model analysis.

Covariates	Sub-hazard ratio	P value	95% Confidence interval	
Age at diagnosis	1.223	0.080	0.976	1.532
Non-MA conditioning	4.968	0.054	0.974	25.333
Graft failure	24.478	0.005	2.637	227.234
Viral infection	4.133	0.034	1.115	15.319
Chronic GVHD	8.710	0.058	0.926	81.921

**Fig 1:**
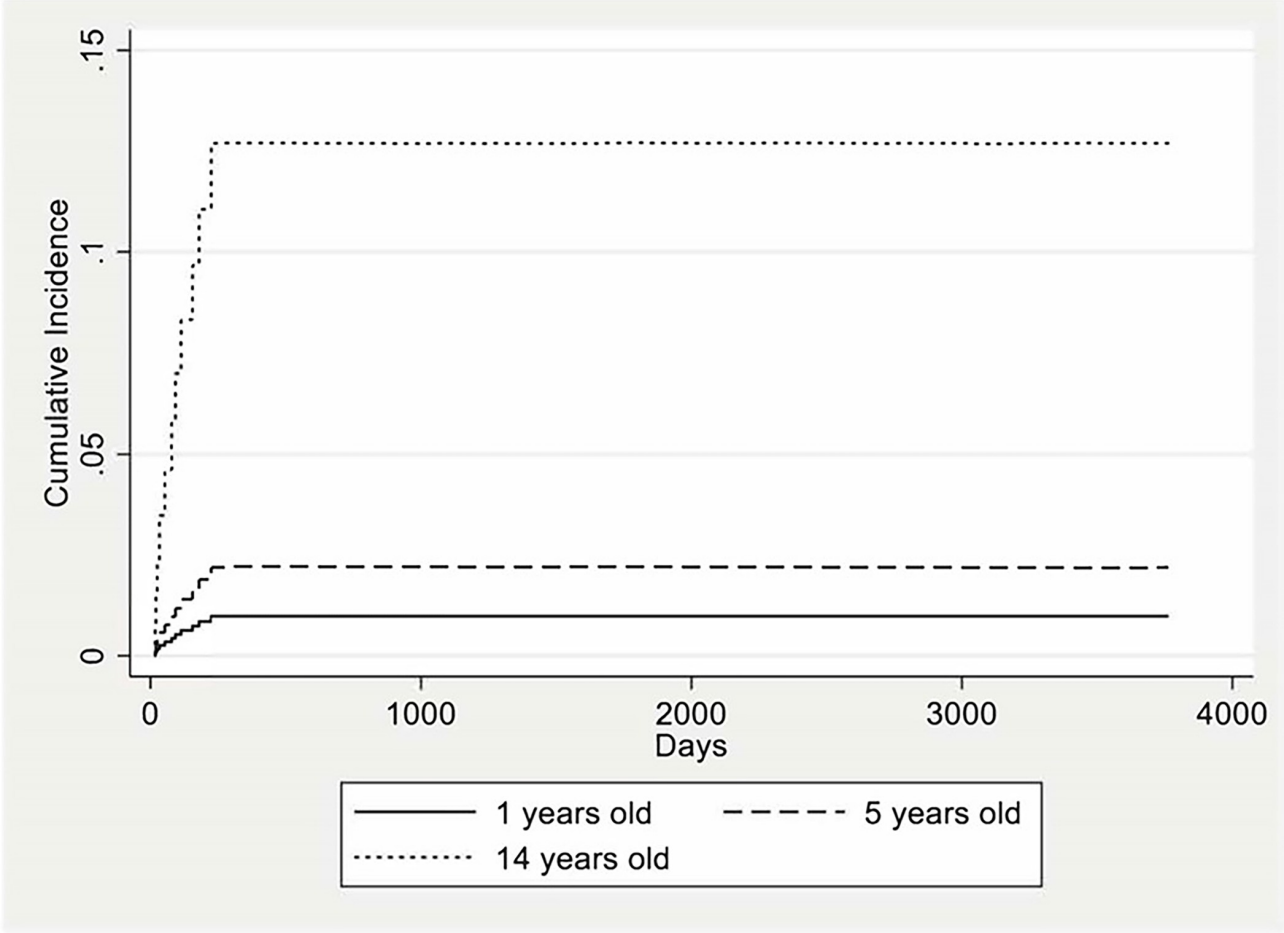
Competing-risk analysis results showing the cumulative incidence functions for infection-related mortality at different ages at diagnosis.

**Fig 2:**
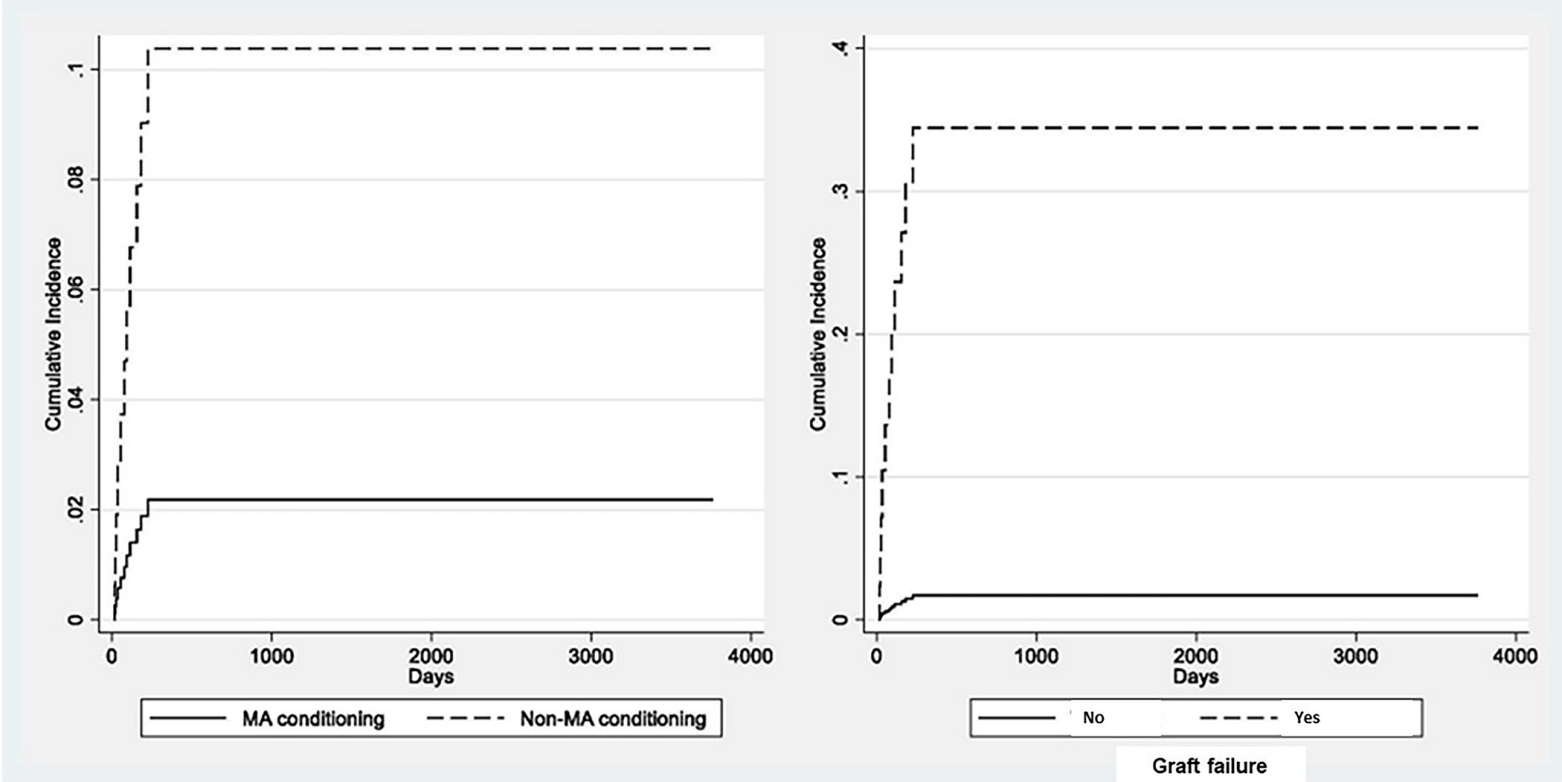
Competing-risk analysis results showing the cumulative incidence functions for infection-related mortality considering the MA conditioning regimen and graft failure (yes/no) as covariates in the model.

Because the estimated subhazard ratio for age at diagnosis is greater than 1, higher age is associated with a higher incidence of IRM controlling for the rest of the covariates, and the fact that relapse and non-IRM can also occur. Being the subhazard ratios of the other covariates greater than 1 indicating a positive association with the incidence of IRM in the presence of competing events (Table 7). The cumulative incidence is shown in Fig 1 whereas the competing-risks model for each covariate is shown in Fig 2.

## Discussion

This study was conducted retrospectively to identify the infectious complications that occurred in pediatric patients treated with allogeneic HSCT over a 10-year period at one of Mexico’s largest hospitals. A cohort of 99 pediatric patients was identified. An average of 10 transplants per year were performed in our unit because of a lack of resources. The conditioning regimen was mainly MA, donor-related, and with peripheral blood as the origin of the HSCs. In our hospital, haploidentical transplantation started in 2016, and a total of 18 procedures have been performed so far, which made a comparison between groups difficult. However, given the lack of studies of this type in our country, we sought to identify the frequency and type of infections, infection-related mortality, and the associated factors in this population.

A total of 136 infectious episodes were recorded for this cohort of 99 children. Bacterial infections predominated (76.4%). These findings are similar to those of other authors such as the 2017 study by Miller et al. [27] who reported 70% of bacterial infections in patients treated with HSCT. In the present study, infections by Gram-positive bacteria were more prevalent. This was also reported in 2013 by Srinivasan et al. [20] in their study of 759 pediatric patients in which Gram-positive bacteria were isolated significantly more frequently than Gram-negative bacteria [4]. The 2016 study of 499 patients by Young et al. [28] recorded 1,347 episodes of infection, 470 of which involved Gram-positive microorganisms. This represented 34.9% of the total number of infectious events, which is slightly lower than the 38.2% observed in the present study. Most studies have reported a predominance of Gram-positive bacteria in children who received HSCT [3, 11, 29], as opposed to adult patients, for whom infections by Gram-negative infectious agents are most prevalent [30]. As in other studies, we found that bacterial infections occurred mainly in the early stage of transplantation (before day 57) [5, 11].

The main Gram-positive bacteria isolated from patients included in the present study were *Staphylococcus epidermidis*, *Enterococcus faecalis*, *and Enterococcus faecium*, and the main Gram-negative bacterium was *Escherichia coli*. This is similar to the types reported in the 2020 study by Akinboyo et al. [29] of 1,294 patients, which found that the main bacteria isolated were *Enterococcus faecium*, *Enterococcus faecalis*, and *Escherichia coli*. Among the factors that could explain the greater frequency of these types of infections are the rupture of natural barriers by the MA conditioning regimen, placement of central and peripheral venous catheters, or inadequate handling of the patient.

The mortality rate because of bacterial infection in the present study was 17.2% (n = 10). This rate is highly variable in the literature (4–37%), and this variability is attributed to multiple risk factors [23–31].

The frequency of fungal infections was 8.1% (n = 11). In patients who died, Candida albicans was the most commonly isolated fungus (2.9%; n = 4). The overall frequency of fungal infection was lower in our study than in other studies. For example, frequencies of 5.8% were reported in 2019 by Lehrnbecher [31], 12% by Akimboyo et al. [29], and 12% by Hol et al. [32]. However, the most frequently found fungi in all studies were *Candida* and *Aspergillus*. The periods when these were most frequently observed were before the engraftment, attributed to neutropenia, and after the graft, attributed to GVHD and high doses of steroid [14, 15, 32].

In our study, 10% (4/40) of all deaths after transplantation was associated with a fungal infection, a rate that is similar to the previously reported in other studies. However, the importance of invasive fungal infection relates to the high mortality rate despite the availability of improved antifungal agents. In a 2020 study by Styczyński et al. [23] of 55,668 deaths from a total of 114,491 patients who received allogeneic HSCT, the mortality associated with fungal infection was 10.6%. In the report by Gomez et al. 30 of 23 patients with allogeneic HSCT, 61% died from proven or probable invasive fungal infection [33]. Similarly, Simms-Waldrip et al. reported that 67.6% of deaths in their study were caused by fungal infection [15].

The frequency of viral infections in our study was 15.5%, and the most common agents were CMV and BK virus. This frequency is lower than that reported earlier [3, 4, 8, 34–36]. This may reflect that the population in our study is over-represented by patients who received allogeneic-type HSCT with a high related donor/receptor compatibility (10/10). In this regard, it has been reported that the rates of infection by BK virus and CMV reactivation are significantly lower in these groups. Particularly, in the study by Ersoy et al. (2023) [37] the frequency of BK virus infection was 9.2% in the high donor compatibility whereas the frequency was higher in the mismatch (9.7%) and the haploidentical (19.3%) groups. Similar results were reported by Atay et al. (2018) [36] where the frequency of BK virus infection was lower in the high donor compatibility group (31%) than in the mismatch and haploidentical groups (37.5% and 38.7%, respectively). In the present study, the BK virus infection was only present in the haploidentical group. Noteworthy, it could be explained by the lower immunosuppression in the group with high donor compatibility in comparison with the mismatch and haploidentical groups. In relation to the CMV prevalence in our study, we observed a low frequency in the high donor compatibility group (6.7%) and higher frequencies in the mismatch and haploidentical groups (18.1% and 16.6%, respectively) as it has been mentioned by other researchers [36]. One limitation of the present research is that we cannot analyze the frequency of other relevant virus such as the norovirus which contributes to GVHD and the adenovirus which plays a role in paediatric mortality within this patient population [38]. These are not routinely investigated in our institution.

The mortality rate for viral infection as a primary cause was 31.6% (n = 6/19) in our study, which is higher in comparison to other reports (12%) [23, 35, 39]. However, it is lower than the reported in the study by Lindsay et al., where among infection-related deaths, 13% were attributed to bacterial infections, 26% to fungal, and 45% to viral infections [40].

Most studies have reported that several factors can increase the risk of infections such as allogeneic HSCT, unrelated donor, MA conditioning regimen, HSCs origin, pretransplant CMV status of the donor and recipient, and presence of GVHD, hospital center, and time of transplant [20, 27–29]. However, few studies have reported older age as a risk factor for infection. In the present study, we found the highest risk of infection in children >10 years of age. This may be related to the difficulty for patients of this age group to perform the preventive measures effectively by themselves, in contrast to children <10 years of age who are cared for mainly by their caregivers who may be more meticulous when performing these measures.

Interestingly, Srinivasan et al. [20] found that the presence of GVHD plus age >10 years increased the risk of fungal infection. Akinboyo et al. [29] reported an increased risk of bloodstream infections with age (RR = 1.03; 95% CI: 1.01–1.05). In another study, Düver et al. [18] found older age to be an important risk factor for viral infection and attributed this primarily to an increased seroprevalence with age and slower thymic recovery in older children.

Another factor identified in the multivariate analysis in the present study was the presence of GVHD as a risk for developing a viral infection after HSCT (aHR = 5.36; 95% CI: 1.62–17.68). As reported consistently by others, the presence of GVHD increases the risk of bacterial, fungal, and viral infections [17, 20, 27, 28]. GVHD is also associated with the risk of viral infection because of its effects on cellular immunodeficiency, which may increase the risk of infection with latent viruses such as CMV and EBV [9, 10]. In our study, two children died as a consequence of pneumonia by CMV, one with acute and the other with chronic GVHD.

Elevated risk for bacterial and viral infection was noted for patients given the MA conditioning regimen, although the confidence intervals were not precise. These observations are similar to those reported in other studies in which a higher risk of infection was found in patients receiving this type of conditioning during the HSCT procedure [11–15].

The documented infection rate was higher than in other places, which should be more associated with the socio-economic state of Mexico versus the developed countries.

In the analysis of the factors associated with infection-related mortality, we found that the MA conditioning regimen was a protective factor. It has been reported that patients receiving an MA regimen achieve a faster graft, which decreases the risk of infection and associated mortality. In this regard, in the present study we observed that those patients who received a non-MA conditioning regimen, had a higher frequency of graft failure (33.3%) which was also associated with a high risk of IRM (OR = 2.78; 95% CI: 2.87–40.49; p = 0.001) in comparison to patients who received a MA conditioning regimen where the graft failure frequency was 12.8%. This finding could explain our results and it is consistent with previous reports where the use of a non-MA conditioning regimen has been reported as a risk factor for mortality after pediatric HSCT [41–43].

Moreover, in the competing risk analysis the MA conditioning regimen and graft status resulted as important covariates for IRM. This is consistent with the literature reviewed for the present research [41–43] and they could be considered as covariates in the multivariate models for identifying risk factors for IRM in pediatric allogeneic HSCT future investigations.

The mortality rate from infection as a primary cause in our study (30.0%) is within the range reported by other studies (16–47.9%) [23, 26–28]. However, we acknowledge that the rate of deaths due to disease relapse after the transplant (52.5%; n = 21/40) was high. In a study of 114,491 patients with HSCT published in 2020, Styczyński et al. reported that 55,668 patients had died and 22,518 (42.9%) had died from disease relapse [23]. In that study population, the origin of HSCs was mainly peripheral blood. Although they have a faster platelet graft, patients who receive high doses of CD34+ have lower overall survival and a higher probability of disease relapse than those who do not receive high doses of CD34+ [44]. This knowledge will lead us to review and improve our program.

Our study had several limitations: This was performed in a single institution; however, we consider that our results are validated taking into account that our institution is one of the biggest as well as an institution of reference in our country. Additionally, this is aninstitution that attends to patients from all over the country. Additionally, some pathogens or characteristics of antibiotic resistance were not found because their detection is not done as routine, in that way all results were not available for conclusive results. A limitation of our study was that previous information about bacterial detection was not available to Us. Our work was based on a retrospective analysis of the results obtained in cultures, performed during the febrile episodes, which were the reason we could not associate previous colonization of our patients with our detection in blood during the febrile episode.

## Conclusions

The frequencies of infections and infection-related mortality in this pediatric population of allogeneic HSCT recipients in Mexico appear to be similar to those reported in other studies. Our results suggest an association of Graft failure with a high risk of mortality after allogeneic HSCT.

## Supporting information

S1 Data Database Transplant Feb 2023 without identifiers(2).sav. The study analysis database has been included.
(SAV)

S1 Table Characteristics of the transplant procedure. S1 Table displays the characteristics of the transplant procedure.
(DOCX)

S2 Table Outcomes of the transplant procedure. Information about the outcomes of the transplant procedure.
(DOCX)

S3 Table Complications attributed to infectious events (n = 136). The specific type of infections, complications and outcomes after the development of infections.
(DOCX)

## References

[1] Goldman J.; Schmitz N.; Niethammer D.; Gratwohl A. Allogeneic and autologous trasplantation for haematological diseases, solid tumors and immune disorders: current practice in Europe in 1998. Bone Marrow Transpl. 1998,21:1–7 10.1038/sj.bmt.1701089 9486487

[2] Juric M.K.; Ghimire S.; Ogonek J.; Milestones of Hematopoietic Stem Cell Transplantation–From First Human Studies to Current Developments. Front Immunol. 2016,7:1–16. 27881982 10.3389/fimmu.2016.00470 PMC5101209

[3] Styczynski J.; Czyzewski K.; Wysocki M.; Gryniewicz-Kwiatkowska O.; Kolodziejczyk-Gietka A.; et al. Increased risk of infections and infection-related mortality in children undergoing haematopoietic stem cell transplantation compared to conventional anticancer therapy: a multicenter nationwide study. Clin Microbiol Infect. 2016,179.e1–179.e10. 26493843 10.1016/j.cmi.2015.10.017

[4] Srinivasan A.; Wang C.; Srivastava D.K.; Timeline, Epidemiology, and Risk Factors for Bacterial, Fungal, and Viral In-fections in Children and Adolescents after Allogeneic Hematopoietic Stem Cell Transplantation. Biol Blood Marrow Transplant. 2013,19:94–101 doi: 10.1016/j.bbmt.2012.08.012 22922523 PMC3554248

[5] Srinivasan A.; McLaughlin L.; Wang C.; Srivastava D.K.; Shook D.R.; Leung W.; Hayden R.T. Early infections after autologous hematopoietic stem cell transplantation in children and adolescents: the St. Jude experience. Transpl Infect Dis. 2014,16:90–97 doi: 10.1111/tid.12165 24256514 PMC4003497

[6] Linder K.A.; McDonald P.J.; Kauffman C.A.; Revankar S.G.; Chandrasekar P.H.; Miceli M.H. Infectious Complications After Umbilical Cord Blood Transplantation for Hematological Malignancy. Open Forum Infect Dis. 2019,22:ofz037. doi: 10.1093/ofid/ofz037 30815505 PMC6386816

[7] Hovi L.; Saarinen-Pihkala UM.; Vettenranta K.; Saxen H. Invasive fungal infections in pediatric bone marrow transplant recipients: single center experience of 10 years. Bone Marrow Transpl. 2000,26:999–1004 doi: 10.1038/sj.bmt.1702654 11100280

[8] Arellano-Galindo J.; Vázquez-Meraz E; Jiménez-Hernández E.; Velazquez-Guadarrama N.; Mikeler E.; Hamprecht K.; et al. The role of cytomegalovirus infection and disease in pediatric bone marrow transplant recipients in Mexico City in the context of viral drug resistance. Pediatr Transplant. 2010, 38(3):219–228. doi: 10.1111/j.1399-3046.2010.01419.x 21199205

[9] Storek J. Immunological reconstitution after hematopoietic cell transplantation its relation to the contents of the graft. Expert Opin. Biol. Ther. 2008, 8:583–597. doi: 10.1517/14712598.8.5.583 18407763

[10] Ogonek J.; Kralj J.M.; Ghimire S.; Varanasi-Pavankumar R.; Holler E.; Greinix H.; et al. Immune Reconstitution after Allogeneic Hematopoietic Stem Cell Transplantation. Front Immunol 2016, 7:507. doi: 10.3389/fimmu.2016.00507 27909435 PMC5112259

[11] Bock A.M.; Cao Q.; Ferrieri P.; Young J.A.; Weisdorf D.J. Bacteremia in Blood or Marrow Transplantation Patients: Clinical Risk Factors for Infection and Emerging Antibiotic Resistance. Biol Blood Marrow Transplant 2013;19:102–108. doi: 10.1016/j.bbmt.2012.08.016 22940054

[12] Kedia S.; Acharya P.S.; Mohammad F, Farhan M.; Nguyen H.; et al. Infectious Complications of Hematopoietic Stem Cell Transplantation. J Stem Cell Res Ther. 2013,S3.

[13] Donnelly J. P.; Chen S. C.; Kauffman C. A., Steinbach W. J.; Baddley J. W.; Verweij P. E.; et al. Revision and update of the consensus definitions of invasive fungal disease from the European Organization for Research and Treatment of Cancer and the Mycoses Study Group Education and Research Consortium. Clin Infect Dis 2020,71(6): 1367–1376. doi: 10.1093/cid/ciz1008 31802125 PMC7486838

[14] Poutsiaka D.D.; Munson D.; Price L.L.; Chan G.W.; Snydman D.R. Blood stream infection (BSI) and acute GVHD after hematopoietic SCT (HSCT) are associated. Bone Marrow Transplant. 2011;46:300–307 doi: 10.1038/bmt.2010.112 20479711 PMC3049187

[15] Simms-Waldrip T.; Rosen G.; Nielsen-Saines K.; Alan-Ikeda A.; Brown B.; Moore T. Invasive fungal infections in pediatric hematopoietic stem cell transplant patients. Infect Dis.2015,47:218–24 doi: 10.3109/00365548.2014.985709 25650728

[16] Robenshtok E.; Gafter-Gvili A.; Goldberg E.; Weinberger M.; Yeshurun M.; Leibovici L.; et al. Antifungal prophylaxis in cancer patients after chemotherapy or hematopoietic stem-cell transplantation: systematic review and meta-analysis. J Clin Oncol 2007,25:5471–89. doi: 10.1200/JCO.2007.12.3851 17909198

[17] Boeckh M.; Ljungma P. How we treat cytomegalovirus in hematopoietic cell transplant recipients. Blood. 2009,113:5711–19 doi: 10.1182/blood-2008-10-143560 19299333 PMC2700312

[18] Düver F.; Weißbrich B.; Eyrich M.; Wölfl M.; Schlegel P.G.; Wiegering V. Viral reactivations following hematopoietic stem cell transplantation in pediatric patients A single center 11-year analysis. PLOS ONE, 2020. 15(2):e0228451. doi: 10.1371/journal.pone.0228451 32017805 PMC6999888

[19] Tomblyn M.; Chiller T.; Einsele H. Guidelines for Preventing Infectious Complications among Hematopoietic Cell Transplantation Recipients: A Global Perspective. Biol Blood Marrow Transplant. 2009;15:1143–1238 doi: 10.1016/j.bbmt.2009.06.019 19747629 PMC3103296

[20] Srinivasan A.; Wang C.; Srivastava D.K.; Burnette K.; Shenep J.L.; Leung W.; et al. Timeline, Epidemiology, and Risk Factors for Bacterial, Fungal, and Viral Infections in Children and Adolescents after AllogeneicHematopoietic Stem Cell Transplantation. Biol Blood Marrow Transplant. 2013,19:94–101 doi: 10.1016/j.bbmt.2012.08.012 22922523 PMC3554248

[21] Gerna G.; Lilleri D.; Furione M.; Baldanti F. Management of human cytomegalovirus infection in transplantation: validation of virologic cut-offs for preemptive therapy and immunological cut-offs for protection. New Microbiol. 2011,34:229–54 21811744

[22] Gooley T.A.; Chien J.W.; Pergam S.A.; Hingorani S.; Sorror M.L.; Boeckh M.; et al. Reduced mortality after allogeneic hematopoietic-cell transplantation. N Engl J Med 2010;363:2091–2101 doi: 10.1056/NEJMoa1004383 21105791 PMC3017343

[23] Styczyński J.; Tridello G.; Koster L.; Iacobelli S.; van Biezen A.; van der Werf S.; et al. Death after hematopoietic stem cell transplantation: changes over calendar year time, infections and associated factors. Bone Marrow Transpl 2020;55:126–136. doi: 10.1038/s41409-019-0624-z 31455899 PMC6957465

[24] Yokoe D, Casper C, Dubberke E, Lee G, Muñoz P, Palmore T, et al. Infection prevention and control in health-care facilities in which hematopoietic cell transplant recipients are treated. Bone Marrow Transpl. 2009;44(8):495–507. doi: 10.1038/bmt.2009.261 19861984

[25] von Elm E, Altman DG, Egger M, et al. The Strengthening the Reporting of Observational Studies in Epidemiology (STROBE) statement: guidelines for reporting observational studies. Ann Intern Med. 2007;147:573–577. doi: 10.7326/0003-4819-147-8-200710160-00010 17938396

[26] Fine J.P., Gray R.J. A proportional hazards model for the subdistribution of a competing risk. J Amer Statist Assoc. 1999,94:496–509.

[27] Miller H.K.; Braun T.M.; Stillwell T.; Harris A.C.; Choi S.; Connelly J.; et al. Infectious Risk after Allogeneic Hemato-poietic Cell Transplantation Complicated by Acute Graft-versus-Host Disease. Biol Blood Marrow Transplant. 2017,23:522–528 doi: 10.1016/j.bbmt.2016.12.630 28017733 PMC5551893

[28] Young J.H.; Logan B.R.; Wu J.; Wingard J.R.; Weisdorf D.J.; Mudrick C.; et al. Infections following Transplantation of Bone Marrow or Peripheral-Blood Stem Cells from Unrelated Donors. Biol Blood Marrow Tr. 2016,22:359–370 10.1016/j.bbmt.2015.09.013 PMC4716871 26409243

[29] Akinboyo I.C.; Young R.R.; Spees L.P.; Heston S.M.; Smith M.J.; Chang YC.; Et al. Microbiology and Risk Factors for Hospital-Associated Blood stream Infections Among Pediatric Hematopoietic Stem Cell Transplant Recipients. Open Forum Infect Dis. 2020,7(4):ofaa093. doi: 10.1093/ofid/ofaa093 32284949 PMC7141603

[30] Czyżewski K. Styczyński J, Giebel S, Frączkiewicz J, Salamonowicz M.; Zając‐Spychała O. Age-dependent determinants of infectious complications profile in children and adults after hematopoietic cell transplantation: lesson from the nationwide study. Ann Hematol. 2019,98:2197–2211. 31321454 10.1007/s00277-019-03755-2 PMC6700048

[31] Lehrnbecher T.; Schöning S.; Poyer F.; Georg J.; Becker A.; Gordon K.; et al. Incidence and Outcome of Invasive Fungal Diseases in Children With Hematological Malignancies and/or Allogeneic Hematopoietic Stem Cell Transplantation: Results of a Prospective Multicenter Study. Front Microbiol. 2019,16;10:681. doi: 10.3389/fmicb.2019.00681 31040830 PMC6476895

[32] Hol J.A.; Wolfs T.F.; Bierings M.B.; Lindemans C.A.; Versluys A.B.; Wildt de A.; et al. Predictors of invasive fungal infection in pediatric allogeneic hematopoietic SCT recipients. Bone Marrow Transplant. 2014,49(1):95–101. doi: 10.1038/bmt.2013.136 24121212

[33] Gomez S.M.; Caniza M.; Fynn A.; Vescina C.; Ruiz C.D.; Iglesias D.; et al. Fungal infections in hematopoietic stem cell transplantation in children at a pediatric children’s hospital in Argentina. Transpl Infect Dis. 2018;20(4):e12913. doi: 10.1111/tid.12913 29679436

[34] Gałązka P.; Dziedzic M.; Czyżewski K.; Styczyński J. Differential risk of viral infections in children undergoing complex anticancer therapy or hematopoietic stem cell transplantation. Med Res J 2018;3:127–133

[35] Slade M.; Goldsmith S; Romee R.; DiPersio J.F.; Dubberke E.R.; Westervelt P.; et al. Epidemiology of infections fol-lowing haploidentical peripheral blood hematopoietic cell transplantation. Transpl Infect Dis. 2017,19(1):e12629. doi: 10.1111/tid.12629 28030755 PMC5459579

[36] Atay D.; Akcay A.; Erbey F.; Ozturk G. The impact of alternative donor types on viral infections in pediatric hemato-poietic stem cell transplantation. Pediatr Transplant. 2018,22(2):e13109. doi: 10.1111/petr.13109 29297965 PMC7167794

[37] Ersoy GZ, Bozkurt C, Aksoy BA, Öner ÖB, Aydogdu S, Cipe F, et al. Evaluation of the risk factors for BK virus-associated hemorrhagic cystitis in pediatric bone marrow transplantation patients: Does post-transplantation cyclophosphamide increase the frequency? Pediatr Transplant 2023 Feb:27 (1):e 14364. doi: 10.1111/petr.14364 Epub 2022 Jul 19. . 35851981

[38] Robles JD; Cheuk DK, Ha SY, Chiang AK, Chan GC. Norovirus infection in pediatric hematopoietic stem cell trasnplantation recipients: incidence, risk factors, and outcome. Biol Blood Marrow Transplant. 2012 Dec;18(12):1883–9. doi: 10.1016/j.bbmt.2012.07.005 Epub 2012 Jul 10. . 22796532

[39] Tsoumakas K.; Giamaiou K.; Goussetis E.; Graphakos S.; Kossyvakis A.; Horefti E.; et al. Epidemiology of viral infections among children undergoing hematopoietic stem cell transplant: Α prospective single‐center study. Transpl Infect Dis. 2019,21:e13095. 30993823 10.1111/tid.13095

[40] Lindsay J., Kerridge I., Wilcox L., Tran S., O’Brien T. A., Greenwood M., et al. (2021). Infection-Related Mortality in Adults and Children Undergoing Allogeneic Hematopoietic Cell Transplantation: An Australian Registry Report. Transplantation and Cellular Therapy, 27(9), 798–e1. doi: 10.1016/j.jtct.2021.05.028 34111574

[41] Satwani P.; Jin Z.; Duffy D. Transplantation-Related Mortality, Graft Failure, and Survival after Reduced-Toxicity Conditioning and Allogeneic Hematopoietic Stem Cell Transplantation in 100 Consecutive Pediatric Recipients. Biol Blood Marrow Transplant. 2013,19(4):552–61. doi: 10.1016/j.bbmt.2012.12.005 23253557

[42] Olsson R.; Remberger M.; Schaffer M.; Berggren D.M.; Svahn B.M.; Mattsson J.; et al. Graft failure in the modern era of allogeneic hematopoietic SCT. Bone Marrow Transplant. 2013,48(4):537–543. doi: 10.1038/bmt.2012.239 23222384

[43] Cluzeau T.; Lambert J.; Raus N.; Dessaux K.; Absi L.; Delbos F.; et al. Risk factors and outcome of graft failure after HLA matched and mismatched unrelated donor hematopoietic stem cell transplantation: a study on behalf of SFGM-TC and SFHI. Bone Marrow Transplant. 2016, 51(5):687–691. doi: 10.1038/bmt.2015.351 26855158

[44] Remberger M.; Törlén J.; Ringdén O.; Engström M.; Watz E.; Uhlin M.; et al. Effect of Total Nucleated and CD34(+) Cell Dose on Outcome after Allogeneic Hematopoietic Stem Cell Transplantation. Biol Blood Marrow Transplant. 2015,21:889–893. doi: 10.1016/j.bbmt.2015.01.025 25662230

