# Supplementary material for: Infections and risk factors for infection-related mortality after pediatric allogeneic hematopoietic stem cell transplantation in Mexico: A single center retrospective study
Source: PLoS One. 2023 Sep 29;18(9):e0284628. doi: 10.1371/journal.pone.0284628 (PMC10540957; doi:10.1371/journal.pone.0284628)
Supplement: S1 Table — displays the characteristics of the transplant procedure. (DOCX) [file pone.0284628.s002.docx]

**Supplementary Table 1.** Characteristics of the transplant procedure

**Variables n (%) Med Min/Max**

**Conditioning regimen**

Myeloablative 78 (78.8)

Non-myeloablative 21 (21.2)

**Conditioning schemes**

Busulfan, cyclophosphamide 71 (71.7)

Cyclophosphamide, fludarabine with ATG 7 (7.1)

Cyclophosphamide, with ATG 11 (11.1)

Cyclophosphamide, TBI 6 (6.1)

Other 4 (4.0)

**Donor type**

Related 80 (80.8)

Unrelated 19 (19.2)

**Compatibility status**

Related, identical 59 (59.6)

Related, mismatch 4 (4.0)

Unrelated, identical 4 (4.0)

Unrelated, mismatch 14 (14.1)

Haploidentical 18 (18.2)

**Origin of HSCs**

Peripheral blood 80 (80.8)

Umbilical cord 19 (19.2)

**GVHD prophylaxis**

Cyclosporine 18 (18.2)

Cyclosporine + methotrexate 20 (20.2)

Cyclosporine + MMF 22 (22.2)

Cyclosporine + MMF + cyclophosphamide 33 (33.3)

Other 6 (6.0)

**CMV serological status of the recipient (pretransplant)**

Negative 15 (15.2)

Positive 84 (84.8)

**CMV serological status of the donor (pretransplant)**

Negative 14 (14.1)

Positive 85 (85.9)

**Anti-infective prophylaxis**

Ciprofloxacin, fluconazole, acyclovir 19 (19.2)

Cefepime, voriconazole, acyclovir 80 (80.8)

Min, minimum; Max, maximum; ATG, anti-thymocyte globulin; TBI, total body irradiation; HSCs, hematopoietic stem cells; GVHD, graft-versus-host disease; MMF, mycophenolate mofetil; CMV, cytomegalovirus
