# Supplementary material for: Infections and risk factors for infection-related mortality after pediatric allogeneic hematopoietic stem cell transplantation in Mexico: A single center retrospective study
Source: PLoS One. 2023 Sep 29;18(9):e0284628. doi: 10.1371/journal.pone.0284628 (PMC10540957; doi:10.1371/journal.pone.0284628)
Supplement: S2 Table — Information about the outcomes of the transplant procedure. (DOCX) [file pone.0284628.s003.docx]

**Supplementary Table 2. Outcomes of the transplant procedure**

Variables n (%) Median Min/Max

**Graft achievement**

Yes 80 (80.8)

No 19 (19.2)

**Time at graft achievement (days) 14 7/34**

**Graft lost**

Yes 17 (17.2)

No 82 (82.8)

**Time at graft lost (days) 133 34/1482**

**Acute GVHD 46 (46.5)**

Grade I 27 (58.7)

Grade II 9 (19.6)

Grade III 5 (10.9)

Grade IV 4 (8.7)

Different Grades 1 (2.1)

**Affected organs**

Skin 23 (50.0)

Intestine 2 (4.3)

Liver 5 (10.9)

Skin + intestine 4 (8.7)

Skin + intestine + liver 5 (10.9)

Skin + liver 4 (8.7)

Other 3 (6.6)

**Chronic GVHD** 12 (12.8)

**Outcomes**

Alive 59 (59.6)

Deaths 40 (40.4)

**Causes of death**

Deaths due to disease relapse 21 (52.5)

Other causes of death 7 (17.5)

Infection Related Mortality 12 (30.0)

**Time of follow-up until death(days) 250 81/2045**

Min= minimum, Max= maximum, GVHD= graft-versus-host disease
