# Supplementary material for: Infections and risk factors for infection-related mortality after pediatric allogeneic hematopoietic stem cell transplantation in Mexico: A single center retrospective study
Source: PLoS One. 2023 Sep 29;18(9):e0284628. doi: 10.1371/journal.pone.0284628 (PMC10540957; doi:10.1371/journal.pone.0284628)
Supplement: S3 Table — The specific type of infections, complications and outcomes after the development of infections. (DOCX) [file pone.0284628.s004.docx]

**Supplementary Table 3.** Complications attributed to infectious events (n=136)

**Types of infection n (%) Median Max/Min**

**Bacterial infections 104 (76.4)**

Complications 58 (55.7)

Loss of venous access 29 (50.0)

Sepsis 18 (31.0)

Septic shock 10 (17.2)

Vulvar abscess 1 (1.8)

**Fungal infections 11 (8.0)**

Complications 6 (54.5)

Septic shock 3 (50.2)

Respiratory failure 1 (16.6)

Loss of venous access 1 (16.6)

Sepsis 1 (16.6)

**Viral infections 21 (15.4)**

Complications 10 (47.6)

GVHD 4 (40.0)

Hemorrhagic cystitis 2 (20.0)

Gastrointestinal bleeding 1 (10.0)

Pneumonia/respiratory failure by CMV infection 2 (20.0)

Transient erythroblastopenia 1 (10.0)

**Outcomes**

Alive 59 (59.6)

Deaths 40 (40.4)

Causes of death

Deaths due to disease relapse 21 (52.5)

Other causes of death 7 (17.5)

Infection Related Mortality 12 (30.0)

Time of follow-up until death(days) 250 81/2045

GVHD= graft-versus-host disease, CMV= Cytomegalovirus
